# Green Processing Technology of Meat and Meat Products

**DOI:** 10.3390/foods12122356

**Published:** 2023-06-13

**Authors:** Changyu Zhou, Jinxuan Cao

**Affiliations:** 1 College of Food Science and Pharmaceutical Sciences, Ningbo University, Ningbo 315211, China; zhouchangyu@nbu.edu.cn; 2 School of Food and Health, Beijing Technology and Business University, Beijing 102401, China

Consumers are increasingly demanding higher quality meat products. However, meat products manufactured using traditional processing procedures are often perceived as unhealthy or hazardous foods, due to the significant amounts of sodium chloride added, the excessive accumulation of harmful substances, and the abnormal growth of spoilage microorganisms. Green processing is a new concept aiming to meet the challenges of the 21st century, and should be the result of a whole chain of values to protect both the environment and consumers. In the meantime, it will hopefully drive industries to be more ecologic, economic, and innovative. Green technologies show great potential in decreasing or preventing the formation of hazardous substances, increasing the shelf life, and maintaining the sensory attributes and nutritional qualities of meat products during the manufacturing of food. Thus, this topic will discuss the effect of green processing on the mechanism of quality development of meat and meat products from the perspective of chemical and biochemical composition and structure changes in molecules during the processing of meat products, as well as the chemistry related to the major components of meat products, and their nutritional, physiological, sensory, flavor, and microbiological properties.

This Special Issue is made up of eighteen research articles aiming at providing up-to-date research on the abovementioned aspects related to raw meat, by-products, meat products, and advances made in meat quality assessment. The different works can be assigned to four distinct groups. One portion of these studies focused on the quality assessment of raw meat; another portion of these studies investigated the antioxidant, gelling, and emulsion properties of proteins; another portion characterized the spoilage of meat and meat products; and the remaining portion mainly evaluated the flavor and texture of processed meat products.

Regarding the quality assessment of raw meat, Cao et al. [1] highlighted the effects of low-temperature storage on the sensory, physicochemical properties, and microstructure of fresh beef, and demonstrated that ice temperature storage and micro-frozen storage both showed great potential in improving the freshness and extending the shelf life of fresh beef. Duan et al. [2] investigated the effect of cooking methods on the edible and nutritional quality of fresh pork, and Tang et al. [3] discussed the efficiency in the simultaneous measurement of fat and moisture contents of pork using low-field nuclear magnetic resonance. Wang et al. [4] highlighted the role of intramuscular connective tissue on structural shrinkage and water loss during the cooking of porcine muscles. Li et al. [5] reported the advantage of pressure shift freezing on the quality of largemouth bass during frozen storage.

Green processing has a significant effect on the functional properties of meat proteins, and on decreasing or preventing the spoilage of meat products. The improvements in the gelling, rheology, and emulsion properties of meat protein were highlighted using typical green technologies, including ultrasonication, high-pressure homogenization, and isoelectric solubilization/precipitation [6,7,8]. Several studies also demonstrated that protein modification also played a role in developing the antioxidant properties of meat protein [9] and the color stability of meat products [10]. Spoilage is a common phenomenon during the storage of meat and the processing of meat products and *Clostridium* and *Pseudomonas* are the key genera involved in this process. *Pseudomonas lundensis* exhibited the strongest spoilage potential during the storage of chilled chicken [11]; *Clostridium* was mainly responsible for the spoilage in deeply spoiled ham [12]. The combination of mild heat and lactic acid showed a great potential to inactivate the spoilage microorganisms including *Clostridium perfringens* [13]. Furthermore, *Pseudomonas* was also inhibited by the chitosan/collagen peptides/cinnamon bark essential oil composite coating [14].

Texture and flavor are the key parameters of fermented and spiced meat products. The addition of halogen and spicy flavors showed a positive effect for the relative content of alkanes and ketones during the processing of a Midu pork roll [15]. Zhang et al. [16] characterized the effect of processing stage on the physicochemical properties and volatile flavor components of dry-cured donkey leg, and demonstrated that aldehydes, esters, alkanes, and alcohols were more abundant in the final products. Wang et al. [17] demonstrated that ultrasound combined with sous-vide cooking was closely related to the improvements in the textural quality of spiced beef. Wang et al. [18] demonstrated that the treatment of high pressure at 50 °C significantly improved the textural parameters of Chinese traditional pig trotter with soy sauce. However, Zhang et al. [14] found that the treatment of chitosan/collagen peptides/cinnamon bark essential oil composite coating reduced some volatile compounds such as alcohols, aldehydes, ketones, and acetate during the processing of dry-aged beef, but the treatment only had a slight influence on the quality of dry-aged beef. In summary, this Special Issue provides insights into the recent achievements in the storage and processing of meat and meat products using green technologies.
